# Supplementary material for: Curated and harmonised transcriptomics datasets of interstitial lung diseases
Source: Data Brief. 2025 Oct 14;63:112139. doi: 10.1016/j.dib.2025.112139 (PMC12581653; doi:10.1016/j.dib.2025.112139)
Supplement: Supplementary file 1 [file mmc1.zip › Supplementary_material/RNA-seq/fastQC reports/GSE169500/SRR14057121_fastqc.html]

SRR14057121.fastq.gz FastQC Report 

FastQC Report

Mon 5 Feb 2024  
SRR14057121.fastq.gz

## Summary

- Basic Statistics
- Per base sequence quality
- Per sequence quality scores
- Per base sequence content
- Per sequence GC content
- Per base N content
- Sequence Length Distribution
- Sequence Duplication Levels
- Overrepresented sequences
- Adapter Content

## Basic Statistics

| Measure | Value |
| --- | --- |
| Filename | SRR14057121.fastq.gz |
| File type | Conventional base calls |
| Encoding | Sanger / Illumina 1.9 |
| Total Sequences | 12772926 |
| Sequences flagged as poor quality | 0 |
| Sequence length | 25-369 |
| %GC | 50 |

## Per base sequence quality

## Per sequence quality scores

## Per base sequence content

## Per sequence GC content

## Per base N content

## Sequence Length Distribution

## Sequence Duplication Levels

## Overrepresented sequences

| Sequence | Count | Percentage | Possible Source |
| --- | --- | --- | --- |
| AGAGCATGACCGATGGATTCCAGTTCGAGTATGGCGGCCAGGGCTCCGAC | 116307 | 0.9105744447278564 | No Hit |
| GGGCCACGAGCTGAGTGCGTCCTGTCACTCCACTCCCATGTCCCTTGGGA | 102231 | 0.8003726006085059 | No Hit |
| CAATATCAAGGAAGGGCAGGCGTGATGGCTTATTTGTTTTGTATTCAATG | 96070 | 0.7521377638921576 | No Hit |
| TTCTTGTAGATGGCTGCTCTAAAAAGACAAATGAATGGGGAAAGACAATC | 88750 | 0.6948290470014467 | No Hit |
| GGGAATGTCTACGTGCGTATGCACGTGGCACTCTCTGCCCGAGGTCCGGG | 85787 | 0.671631543156204 | No Hit |
| ACCAATGTCATAGGGTGCAATATCTACAATAGGTAGTCTCACAGCCTTGC | 85308 | 0.6678814235673173 | No Hit |
| TGGTTGCACGAAACACACTGGGGAATGGAGCAAAACAGTCTTTGAATATC | 71987 | 0.5635905195097818 | No Hit |
| ATGTTCTGGGAGGCCTCGGTGGACATCAGGCGCAGGAAGGTCAGCTGGAT | 70267 | 0.5501245368524017 | No Hit |
| AAGCTCAGGGAGAGCCCTGTTAGGGCCGCCTCTGGCCCTAGTCTCAGACC | 56606 | 0.4431717525021283 | No Hit |
| ACACTGAGGACTCTGTTCCTCCCCTTTCCGCCTAGGGGAAAGTCCCCGGA | 54715 | 0.4283670006386947 | No Hit |
| TGAAGCTCAGGGAGAGCCCTGTTAGGGCCGCCTCTGGCCCTAGTCTCAGA | 42674 | 0.3340972929773491 | No Hit |
| ATGTTCTGGGAGCCTCGGTGGACATCAGGCGCAGGAAGGTCAGCTGGATG | 42154 | 0.330026181941397 | No Hit |
| ACACCCACCGCAACTGTCTGTCTCATATCACGAACAGCAAAGCGACCCAA | 37151 | 0.29085739633972674 | No Hit |
| TGGTTCCTGGCAAGCCCATGTGTGTTGAGAGCTTCTCAGACTATCCACCT | 37095 | 0.2904189689973934 | No Hit |
| ACACTGAGGACTCTGTTCCTCCCCTTCCGCCTAGGGGAAAGTCCCCGGAC | 30078 | 0.23548245719109312 | No Hit |
| GCTGCTACAGGAGAATAGCAGACAGGTATAGTTAAGAAACAACTTTATTT | 29466 | 0.2306910726641648 | No Hit |
| ATTCTTCCTACCCATGAGCATGGAATGTTCTTCCATTTGTTTGTATCCTC | 28008 | 0.21927630364412978 | No Hit |
| TCACACTTCATGATGGAGTTGAAGGTAGTTTCGTGGATGCCACAGGACTC | 24320 | 0.1904027315276077 | No Hit |
| AGCGGTTCCGCTGCCCTGAGGCACTCTTCCAGCCTTCCTTCCTGGGCATG | 21452 | 0.16794898835239475 | No Hit |
| ACACTGAGGACTCTGTTCCTCCCCTTCCGCCTAGGGAAAGTCCCCGGACC | 19531 | 0.1529093646984254 | No Hit |
| TGGCTCTCCCAGAGGCAGAAGGACAATGGCTGTTTCAGGAGCTCTGGGTC | 19180 | 0.1501613647491577 | No Hit |
| TGTTCCCTCTCCTCATGAGATTGGTGAAGAAAGTATTTGGCAAAGTTCTT | 19007 | 0.14880693742373516 | No Hit |
| GGTTATAATCATTGGCAATGTTGTAATCAACAACCCATGTTATTTCTACA | 18969 | 0.1485094331557233 | No Hit |
| AAGAGAAAGCAGTGCAAACCTTCCCGTGGCCGCAAGCGTGGCATCTGCTG | 18955 | 0.14839982632013995 | No Hit |
| TCAACGTACTCCATGCCTGGCAGCTTCATCCCGTACTTGTCCACGCACCA | 18315 | 0.14338922812204502 | No Hit |
| TCTGCAATGCAATCACAATGCCCAAACTAGACCTGCCATTTCTCACACAC | 17928 | 0.14035938202413448 | No Hit |
| AATACCAAGACCTGCTCAATGTTAAGATGGCCCTTGACATTGAGATTGCC | 16808 | 0.13159083517746833 | No Hit |
| TGTTGTGCTAAACGCTGGGAGGGTACAAGTTGGTCATTCCTAAATCTGTG | 16507 | 0.1292342882124268 | No Hit |
| CAGTAGCACCAACGTTGATGAGGTCGTGAGTCAGGGGTCCTAGGAAGCTC | 16358 | 0.12806775831943285 | No Hit |
| TGACTGGTGATGCTGATATTGGAGTGCATCTGAGGCTGTTGTAGAAATAA | 16253 | 0.1272457070525579 | No Hit |
| CAGCATGATTTATAGTCCTTTGGGTATATACCCAGTAATGGGATGGCTGG | 16175 | 0.12663504039716506 | No Hit |
| TGCATATACTGGTTAGTGAGGTGAGCCTGGCGCTCTTCTTTGCGCTGAGC | 15970 | 0.12503008316183778 | No Hit |
| TCTTCAAGGAGAACTACAAACCACTGCTCAAGGAAATAAAAGAGGATACA | 15733 | 0.12317459601660576 | No Hit |
| CTAAGTGCTGTGTTGTCGTTCCCCCTGCTTAAAATAAAGTTGTTTCTTAA | 15029 | 0.11766293799870133 | No Hit |
| TGTCCATGTCTTACTACTTTGACCGCGATGATGTGGCTTTGAAGAACTTT | 15010 | 0.11751418586469536 | No Hit |
| CAGAAGCTCTTTATGTCAAACTTCTCAAGTTGATTGACCTCCAGGTAATA | 14986 | 0.1173262884322668 | No Hit |
| TTTACACCTGCTAGGGTGTTCAAAGGTCAGTGCTATAGAAATTCAGTATC | 14464 | 0.11323951927694562 | No Hit |
| TCTGTCTTCTTCAGTTTCGACTTATCGAATTTCTCGATCTCAGCCATATC | 14433 | 0.1129968184267254 | No Hit |
| AGTGTGGCGATTCCTCAGGGATCTAGAACTAGAAATACCATTGACCCAGC | 14270 | 0.11172068169814811 | No Hit |
| TGTGCCCTGGAAGCGGAATGGAAACTTGCAGGGCTGTCCTTCAGCGTTGC | 14102 | 0.11040539967114818 | No Hit |
| TTCCCCCTGCGCATGCGGGACTGGCTCAAGAACGTCCTGGTCACCCTGTA | 14052 | 0.110013946686922 | No Hit |
| CGGTTGTACTTGCGGATGTAGTGCAGATAGTCTCGGCGGATGACAATGGT | 13735 | 0.1075321347669281 | No Hit |
| GAGAGGGTCACTTCATCTTCTACTCCTCCCTTTATGGCATTGTTGAGCAG | 13711 | 0.10734423733449955 | No Hit |
| ACACTGAGGACTCTGTTCCTCCCCTTTCCGCCTAGGGAAAGTCCCCGGAC | 13442 | 0.10523822027936278 | No Hit |
| GAAAAGTTTGGAAGAGGCAGAGAAATCCTGCTCTCCTCGCCTTCCAGCAG | 13279 | 0.10396208355078547 | No Hit |

## Adapter Content

Produced by FastQC (version 0.11.7)
